# Uteroplacental insufficiency down regulates insulin receptor and affects expression of key enzymes of long-chain fatty acid (LCFA) metabolism in skeletal muscle at birth

**DOI:** 10.1186/1475-2840-7-14

**Published:** 2008-05-18

**Authors:** Daniela Germani, Antonella Puglianiello, Stefano Cianfarani

**Affiliations:** 1 Department of Public Health and Cell Biology, Tor Vergata University, 00133 Rome, Italy

## Abstract

**Background:**

Epidemiological studies have revealed a relationship between early growth restriction and the subsequent development of insulin resistance and type 2 diabetes. Ligation of the uterine arteries in rats mimics uteroplacental insufficiency and serves as a model of intrauterine growth restriction (IUGR) and subsequent developmental programming of impaired glucose tolerance, hyperinsulinemia and adiposity in the offspring. The objective of this study was to investigate the effects of uterine artery ligation on the skeletal muscle expression of insulin receptor and key enzymes of LCFA metabolism.

**Methods:**

Bilateral uterine artery ligation was performed on day 19 of gestation in Sprague-Dawley pregnant rats. Muscle of the posterior limb was dissected at birth and processed by real-time RT-PCR to analyze the expression of insulin receptor, ACCα, ACCβ (acetyl-CoA carboxylase alpha and beta subunits), ACS (acyl-CoA synthase), AMPK (AMP-activated protein kinase, alpha2 catalytic subunit), CPT1B (carnitine palmitoyltransferase-1 beta subunit), MCD (malonyl-CoA decarboxylase) in 14 sham and 8 IUGR pups.

Muscle tissue was treated with lysis buffer and Western immunoblotting was performed to assay the protein content of insulin receptor and ACC.

**Results:**

A significant down regulation of insulin receptor protein (p < 0.05) and reduced expression of ACS and ACCα mRNA (p < 0.05) were observed in skeletal muscle of IUGR newborns. Immunoblotting showed no significant change in ACCα content.

**Conclusion:**

Our data suggest that uteroplacental insufficiency may affect skeletal muscle metabolism down regulating insulin receptor and reducing the expression of key enzymes involved in LCFA formation and oxidation.

## Background

Uteroplacental insufficiency resulting in fetal growth retardation is a common complication of pregnancy and a significant cause of perinatal morbidity and mortality. Epidemiologic studies in humans have shown that impaired intrauterine growth is associated with an increased incidence of insulin resistance, type 2 diabetes, and cardiovascular disease in the adult [1-6]. These observations have led to the hypothesis that metabolic and cardiovascular disease in adulthood arises *in utero*, in part, as a result of changes in the development of key endocrine and metabolic pathways during suboptimal intrauterine conditions associated with impaired fetal growth. This hypothesis has been tested experimentally in a number of species, using a range of techniques to impair fetal growth. Inducing intrauterine growth retardation (IUGR) by placental insufficiency or by undernutrition, stress, or hormone treatment of the mother leads to endocrine and metabolic alterations in the adult offspring in several species [7]. However, the mechanisms by which an abnormal uterine milieu leads to the development of diabetes in adulthood are not known. To investigate potential sequelae of IUGR and the underlying mechanisms, ligation of the uterine arteries in rats has been used as an animal model of uteroplacental insufficiency leading to growth retarded fetuses with a metabolic profile very similar to that of IUGR human fetuses [8,9]. We have recently shown that uteroplacental insufficiency, obtained by uterine artery ligation, which leads to IUGR pups, affects the expression of specific hypothalamic lipid sensing genes such as the CPT1 isoform C, and acetyl-CoA carboxylase (ACC) isoforms alpha and beta [10].

Insulin resistance and type 2 diabetes are characterized by hyperglycemia with hyperinsulinemia, a reduced ability to oxidize fat, and an accumulation of fat within skeletal muscle [11,12]. This increase in muscle fat content is highly associated with insulin resistance [13,14]. Recently, perturbed skeletal muscle insulin signaling has been reported in adult growth restricted rats [15].

In this study we focused on the hypothesis that uteroplacental insufficiency may affect skeletal muscle metabolic pathways altering the expression of insulin receptor and of key enzymes of intramuscular lipid metabolism.

## Methods

### Animal model

Time-dated Sprague-Dawley pregnant rats (Harlan Sprague Dawley, Inc.) were individually housed under standard conditions and allowed free access to standard chow and water. On day 19 of gestation (term is 22 days) the maternal rats were anesthetized with intramuscular injections of xylazine (8 mg/Kg) and ketamime (40 mg/kg) (Sigma-Aldrich, St. Louis, MO), and the abdomen was opened along the midline. Suture was placed around both uterine arteries, then either tied or withdrawn before closing the abdomen [8,9]. Dams recovered quickly from uterine artery ligation (n = 4) and sham procedures (n = 4), and resumed feeding the same day. After recovery, rats had ad libitum access to food and water. The pregnant rats were allowed to deliver spontaneously and at birth pups were weighted and killed by cervical dislocation. Posterior limb skeletal muscle tissue was immediately harvested and frozen in liquid nitrogen and stored at -80°C. 14 sham and 8 IUGR rats from different litters were tested. All procedures complied with Italian regulations for laboratory animal care, according to the guidelines and under supervision of the Animal Technology Station, Interdepartmental Service Center, Tor Vergata University, Rome, Italy.

### Plasma assays

At birth, 14 SHAM and 8 IUGR pups were decapitated, blood was collected and centrifuged at 1900 × *g *at 4°C for 10 min, and plasma was stored at -80°C. Glucose and insulin concentrations were measured.

Glucose was determined using a colorimetric commercial kit (SigmaChemical Co.). Plasma insulin concentrations were measured in duplicate by a rat/mouse insulin ELISA kit, using rat insulin as the standard (Linco Research, St. Charles, MO) according to the manufacturer's instructions. The intraassay CV was 1.2–8.4%, the interassay CV was 6.0–17.9%, and the sensitivity limit was 0.2 ng/mL.

### RNA isolation and cDNA synthesis

Total RNA was extracted using TriPure (Roche Applied Science) according to the manufacturer's instructions and quantified in duplicate using ultraviolet absorbance at 260 nm. Gel electrophoresis confirmed the integrity of the samples. 1 μg RNA, pretreated with RNase free DNase (Invitrogen Co.) was transcribed into the complementary DNA using the High-Capacity cDNA Archive Kit (Applied Biosystems) in a final volume of 50 μl following the manufacturer's protocol. To minimize variation in the reverse transcription reaction, all RNA samples from a single experimental setup were reverse transcribed simultaneously.

### Real-time RT-PCR

We explored the expression of key enzymes that regulate fatty acid metabolism in the muscle. Real-time RT-PCR was performed on an ABI PRISM 7300 Sequencer Detector (Applied Biosystems). PCR primers and TaqMan probes to amplify and detect ACCα, ACCβ, ACS, AMPK, CPT1B, MCD, Insulin receptor and the housekeeping gene 18S were commercially available as inventoried assay (Assay-on-demand Gene Expression Product; Applied Biosystems).

Prior to performing real-time PCR, primer and probe concentrations were determined to demonstrate their specificity and optimal reaction condition. 18S was used as an internal control for differences in cDNA loading. Before the use of 18S as a control, parallel serial dilution of cDNA were quantified to prove the validity of using 18S as an internal control.

The real-time RT-PCR amplification was performed in skeletal muscle tissues from fourteen SHAM and eight IUGR rats. Experiments were performed in triplicate using 96-well tray and optical adhesive covers (Applied Biosystems) in a final reaction mixture of 20 μl containing 3 μl of undiluted cDNA. Real-time PCR was performed using Platinum Quantitative PCR SuperMix-UDG with ROX (Invitrogen Co.). The cycling consisted of 2 min at 50°C, 2 min at 95°C followed by 40 cycles of 95°C for 15 sec and 60°C for 45 sec. Determination of reaction efficiency was routinely used as an internal quality control for adequate assay performance. Crossing of threshold (Ct) values obtained for the target gene were normalized against each individual 18S value which was run in the same well of the real time RT-PCR run. Relative quantification of PCR products was performed using Relative Quantification Study software (Applied Biosystems). Results are expressed in raw relative quantification (RQ) ± standard errorrs.

### Western immunoblotting

Tissues were homogenized in ice with lysis buffer (50 mM Hepes pH 7.4, 150 mM NaCl, 10 mM NaF, 1 mM Na_3_VO_4_, 10% glycerol, 0.5% Triton ×-100, 5 mM EDTA, 10 μl/ml cocktail protease inhibitors). Lysates were clarified by centrifugation at 13,000 g (30 minutes, 4°C), and protein concentration in the supernatant were determined by the Bradford assay (Bio-Rad Laboratories, CA, USA) using bovine serum albumin as a standard. Eighty micrograms of the extracted proteins were separated by SDS-PAGE on 3–8% Tricine gel (Bio-Rad Laboratories, CA, USA) and blotted onto ECL nitrocellulose membrane (Amersham Biosciences UK, Ltd., Little Chalfont, Buckinghamshire, UK). The filter was blocked with 5% non-fat dry milk in TBS-0.1% Tween 20 and then incubated with ACC rabbit polyclonal antibody (Cell Signaling Technology Inc. Danvers, MA) and insulin-Rβ rabbit polyclonal antibody (Santa Cruz). After several washes in PBS-0.1% Tween 20, horseradish peroxidase-conjugated secondary antibody (1:5,000) (Amersham) was added for 1 hour at RT. The labelled bands were detected using Amersham ECL western blotting system according to manufacturer's specifications. After protein detection, membranes were stripped with Restore Western Blot Stripping Buffer (Pierce, Rockford IL) and re-blotted with rabbit HRP-conjugated actin antibody (1:1000) (Santa Cruz). Densitometry analysis of bands was performed using a Image Quant 5 software (Molecular Dynamics).

### Statistical analysis

Statistical analysis was performed using Sigma Plot for Windows Version 13.0 (SPSS, Inc, Chicago, IL, USA). Differences in gene expression between sham and IUGR rats were analyzed with one-way ANOVA. Differences between means from plasma assays and densitometric analyses were assessed by unpaired two-tailed *t *test. Differences were considered statistically significant at *p *< 0.05.

## Results

### Animal weights and metabolic profile

Birth weights of IUGR animals were significantly lower than those of controls (SHAM) (mean weight ± SD: 4.0 ± 0.57 *versus *6.5 ± 0.32 g, *p *< 0.001). No significant differences were observed in blood glucose (63.9 ± 13 *vs *62.9 ± 21.5 mg/dL) and insulin (0.34 ± 0.15 *vs *0.36 ± 0.16 ng/mL) levels.

### Insulin Receptor

**Figure 1:**
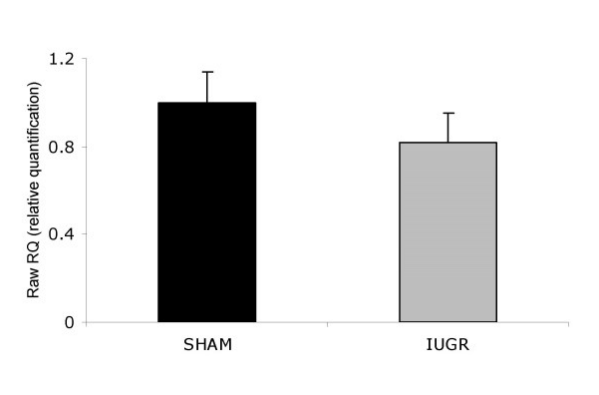
**Insulin receptor mRNA expression in skeletal muscle of SHAM (n = 14) and IUGR rats (n = 8).** Transcripts were measured by real-time RT-PCR using appropriate primers and normalized to 18S mRNA. Data are expressed as relative quantification *vs*. SHAM group (RQ = 1). Bars represent standard errors.

Although a tendency toward a decrease of insulin receptor mRNA expression was noted (20% less), these changes did not achieve statistical significance (Figure 1).

**Figure 2:**
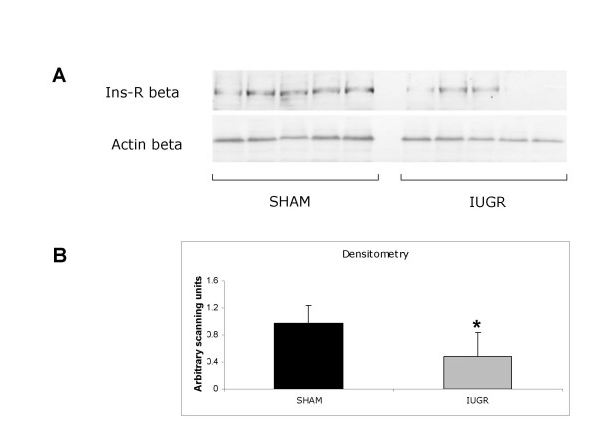
**Insulin receptor beta subunit protein expression in skeletal muscle of SHAM (n = 14) and IUGR rats (n = 8) on day 0.** A, Western immunoblotting analysis; B, densitometric analysis.*P < 0.05.

Immunoblot analysis indicated a significant down regulation of insulin receptor in skeletal muscle of IUGR animals when compared with SHAM (control) (p < 0.05, Figure 2).

### Expression of LCFA metabolism regulatory enzymes

**Figure 3:**
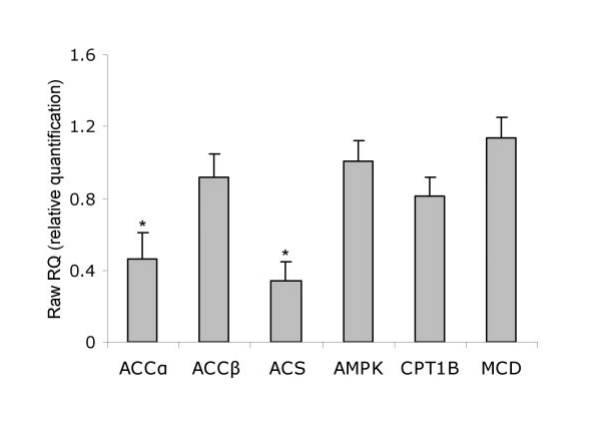
**Expression of acetyl-CoA carboxylase isoenzyme alpha (ACCα) and beta (ACC β), acyl-CoA synthase (ACS), AMP-activated protein kinase (AMPK), carnitine palmitoyltransferase-1 isoenzyme B (CPT1B), malonyl-CoA decarboxylase (MCD), in IUGR rat skeletal muscle.** Transcripts were measured by real-time RT-PCR using appropriate primers and normalized to 18S mRNA. Data are expressed as relative quantification *vs*. SHAM group (RQ = 1). Bars represent standard errors. *P < 0.05.

LCFAs act as nutrient abundance signals in the muscle. The expression of key enzymes that regulate muscle LCFA metabolism was assessed. The expression of ACCα and ACS mRNA levels was significantly reduced (by 54% and 66% respectively, p < 0.05) in skeletal muscle of IUGR rats at birth (Figure 3), whereas no significant differences in the expression of ACCβ, AMPK, and MCD were observed. Expression of muscle specific *CPT1B *was measured by quantitative RT-PCR but no significant difference between IUGR and SHAM pups was found.

**Figure 4:**
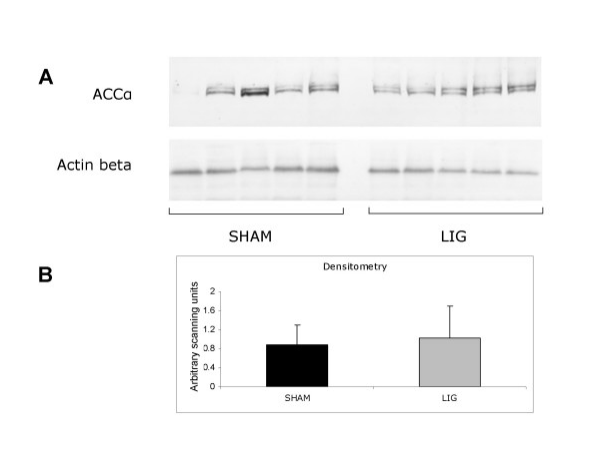
**Acetyl-CoA carboxylase isoenzyme alpha (ACCα) protein expression in skeletal muscle of SHAM (n = 14) and IUGR rats (n = 8) on day 0**. A, western immunoblotting analysis; B, densitometric analysis.

ACCα immunoblotting analysis showed no differences between IUGR and SHAM animals thus suggesting a post-transcriptional regulation (Figure 4). ACS immunoblotting analysis couldn't be performed for unavailability of the specific antibodies.

## Discussion

Uteroplacental insufficiency limits availability of substrates to the fetus and retards growth during gestation, ultimately leading to IUGR. Alterations in the intrauterine milieu have a profound impact on glucose homeostasis in the offspring, culminating in the development of insulin resistance, glucose intolerance and type 2 diabetes in adulthood.

The etiology of insulin resistance within skeletal muscle in the human with type 2 diabetes is multifactorial, involving impairments in hormonal signaling, enzyme and transporter activity, and substrate availability. Previous studies also implicated decreased oxidative capacities of skeletal muscle of human diabetics as contributory to insulin resistance [16].

To investigate the effects of uteroplacental insufficiency on the expression of insulin receptor in muscle, we studied an animal model of intrauterine growth retardation obtained by bilateral uterine artery ligation. In this model, the resulting uteroplacental insufficiency leads to growth retarded fetuses with a metabolic profile very similar to that of IUGR human fetuses [8,9]. These animals exhibit impaired oxidative phosphorylation in skeletal muscle [17], mild peripheral insulin resistance and β-cell secretory defects very early in life but have adequate compensatory insulin secretion for several weeks [18]. However, eventually, β-cell compensation fails, and overt diabetes occurs at age 3–6 months [9]. More recently, it has been described in the same animal model that ligated offspring showed impaired glucose tolerance from the age of 15 weeks as well as elevated glycosylated hemoglobin and corticosterone levels [19].

Our data show for the first time reduced protein levels of insulin receptor in skeletal muscle of IUGR animals. The lack of statistical significance in insulin receptor mRNA expression is probably due to the relative low number of examined samples. Most of previous studies focused on downstream effectors of insulin actions in peripheral tissues without determining insulin receptor expression. In rat model, Ozanne et al. [20] showed that maternal protein restriction leads to muscle insulin resistance. Soleus muscle from growth restricted offspring had similar basal glucose uptakes compared with the control group, but whilst insulin stimulated glucose uptake into control muscle, it had no effect on growth restricted offspring muscle. This impaired insulin action was not related to changes in expression of either the insulin receptor or glucose transporter 4 (GLUT4). However, growth restricted offspring muscle expressed significantly less of the zeta isoform of protein kinase C (PKC ζ) compared with controls. This PKC isoform has been shown to be positively involved in GLUT4-mediated glucose transport. We have used a different model of intrauterine growth restriction based upon uterine artery ligation in which blood flow to the fetus is not ablated but reduced to a similar degree to that observed in human pregnancies complicated by uteroplacental insufficiency. Furthermore, whilst Ozanne et al. [20] studied 15-month-old animals, we investigated rats at birth. The use of only hind limb skeletal muscle might represent a limiting factor since an important difference between type 1 (slow) and type 2 (fast) fibers could exist. However, at birth it is practically impossible to select muscle fibers.

In humans, Jaquet et al. [21] demonstrated that insulin resistance is associated with an impaired regulation of GLUT4 gene expression by insulin in IUGR-born subjects in both skeletal muscle and adipose tissue.

Impairment of muscle fat metabolism is highly associated with insulin resistance. Our study shows for the first time that uteroplacental insufficiency leads to reduced ACS and ACCα mRNA expression in skeletal muscle at birth. In intramuscular lipid metabolism, ACS is the key enzyme for converting free fatty acids into LCFA-CoA. ACC catalyzes the formation of malonyl-CoA, an essential substrate for fatty acid synthesis in lipogenic tissues and a key regulatory molecule in muscle, brain, and other tissues [22]. Three CPT1 isoforms with various tissue distributions and encoded by distinct genes have been identified: liver (CPT1A) [23], muscle (CPT1B) [24], and brain (CPT1C) [25] Cellular levels of malonyl-CoA repress CPT1 activity and decrease LCFA-CoAs oxidation. Therefore, the final effect of reduced expression of both ACS and ACCα would be the decrease of intramuscular LCFAs. This finding is consistent with our recent study in hypothalamus of IUGR rats, showing significant decreased ACCα and ACCβ expression at birth [10]. Taken together these findings suggest that intrauterine programming may affect key enzymes of lipid metabolism at multiple levels. In muscle, however, ACCα protein content was not affected thus suggesting post-transcriptional regulation.

Lipids are implicated in the development of insulin resistance in skeletal muscle. This seems to be linked to an imbalance between lipid supply and lipid oxidation, the latter being related to decreased mitochondrial oxidative capacity in states of insulin resistance [26]. In humans, it has been described that during physiological hyperglycemia with hyperinsulinemia and maintained FFA concentrations (i.e., a condition that mimics the insulin-resistant state), human skeletal muscle malonyl-CoA concentrations are significantly increased and are directly associated with a reduction in LCFA oxidation and functional CPT-1 activity [27].

As LCFA oxidative capacity in mitochondria is low in subjects with insulin resistance [20,28-32], mitochondrial dysfunction and thereby decreased lipid oxidation has been considered to play a key role in the development of insulin resistance. Lane et al. reported an increase in triglyceride levels in IUGR rats [33] Accordingly, a connection between mitochondrial dysfunction, increased intramuscular triacylglycerol levels, and insulin resistance has been described in insulin-resistant offspring of patients with type 2 diabetes [34].

LCFAs may influence glucose metabolism by multiple mechanisms. LCFAs inhibit hexokinase activity [35] thus reducing glucose metabolism. They are also a substrate for the synthesis of ceramide which is increased in muscle of insulin-resistant rats [36]. LCFAs may interfere with insulin signaling by activating protein kinase C. PKC in turn inhibits insulin signaling by phosphorylation of the serine residues on the insulin receptor [37,38] and insulin receptor substrate-1 (IRS-1) [39], thus inhibiting the tyrosine phosphorylation of IRS-1. Patients with type 2 diabetes have increased PKC protein levels in the rectus abdominal muscle [40] and decreased muscle insulin receptor tyrosine kinase activity, which could be restored by phosphatase treatment in vitro, possibly suggesting increased serine phosphorylation of the insulin receptor due to increased PKC activity [37].

We speculate that intrauterine limited supply of substrates secondary to uteroplacental insufficiency may lead to down regulation of insulin receptor in skeletal muscle. We hypothesize that reduced intramuscular content of LCFAs secondary to reduced expression of both ACS and ACC may represent a transient compensatory mechanism to counteract insulin resistance during early postnatal life.

## Conclusion

Uteroplacental insufficiency may affect skeletal muscle metabolism down regulating insulin receptor and reducing the expression of key enzymes involved in LCFA formation and oxidation. We speculate that decreased intramuscular lipid accumulation may represent a transient compensatory mechanism to counteract insulin resistance.

## Abbreviations

ACC: acetyl-CoA carboxylase; ACS: acyl-CoA synthase; AMPK: AMP-activated protein kinase; CPT1: carnitine palmitoyltransferase-1; IUGR: intrauterine growth retardation; LCFA: long-chain fatty acid; MCD: malonyl-CoA decarboxylase.

## Competing interests

The authors declare that they have no competing interests.

## Authors' contributions

DG: participated in the design of the study, carried out the experiments, performed statistical analysis and reviewed the manuscript. AP: carried out the experiments and reviewed the manuscript. SC: conceived the study, supervised the project and drafted the manuscript. He is the corresponding Author of the paper. All Authors read and approved the final manuscript.
